# Multigene Panel Testing Increases the Number of Loci Associated with Gastric Cancer Predisposition

**DOI:** 10.3390/cancers11091340

**Published:** 2019-09-11

**Authors:** Gianluca Tedaldi, Francesca Pirini, Michela Tebaldi, Valentina Zampiga, Ilaria Cangini, Rita Danesi, Valentina Arcangeli, Mila Ravegnani, Raefa Abou Khouzam, Chiara Molinari, Carla Oliveira, Paolo Morgagni, Luca Saragoni, Maria Bencivenga, Paola Ulivi, Dino Amadori, Giovanni Martinelli, Fabio Falcini, Guglielmina Nadia Ranzani, Daniele Calistri

**Affiliations:** 1 Biosciences Laboratory, Istituto Scientifico Romagnolo per lo Studio e la Cura dei Tumori (IRST) IRCCS, 47014 Meldola, Italy; francesca.pirini@irst.emr.it (F.P.); valentina.zampiga@irst.emr.it (V.Z.); ilaria.cangini@irst.emr.it (I.C.); chiara.molinari@irst.emr.it (C.M.); paola.ulivi@irst.emr.it (P.U.); daniele.calistri@irst.emr.it (D.C.); 2 Biostatistics and Clinical Trials Unit, Istituto Scientifico Romagnolo per lo Studio e la Cura dei Tumori (IRST) IRCCS, 47014 Meldola, Italy; michela.tebaldi@irst.emr.it; 3 Romagna Cancer Registry, Istituto Scientifico Romagnolo per lo Studio e la Cura dei Tumori (IRST) IRCCS, 47014 Meldola, Italy; rita.danesi@irst.emr.it (R.D.); mila.ravegnani@irst.emr.it (M.R.); fabio.falcini@irst.emr.it (F.F.); 4 Department of Medical Oncology, Ospedale Infermi, 47923 Rimini, Italy; valentina.arcangeli@auslromagna.it; 5 Department of Biology and Biotechnology, University of Pavia, 27100 Pavia, Italy; raefa.aboukhouzam01@universitadipavia.it (R.A.K.); guglielmina.ranzani@unipv.it (G.N.R.); 6 Institute of Molecular Pathology and Immunology of the University of Porto (Ipatimup), 4200-135 Porto, Portugal; carlaol@ipatimup.pt; 7 Instituto de Investigação e Inovação em Saúde (i3S), University of Porto, 4200-135 Porto, Portugal; 8 Faculty of Medicine of the University of Porto (FMUP), 4200-319 Porto, Portugal; 9 Department of General Surgery, Morgagni-Pierantoni Hospital, 47121 Forlì, Italy; morgagni2002@libero.it; 10 Pathology Unit, Morgagni-Pierantoni Hospital, 47121 Forlì, Italy; luca.saragoni@auslromagna.it; 11 Unit of Upper GI Surgery, University of Verona, 37126 Verona, Italy; mariabenci@hotmail.it; 12 Department of Medical Oncology, Istituto Scientifico Romagnolo per lo Studio e la Cura dei Tumori (IRST) IRCCS, 47014 Meldola, Italy; dino.amadori@irst.emr.it (D.A.); giovanni.martinelli@irst.emr.it (G.M.)

**Keywords:** stomach neoplasms, cancer predisposition, *CDH1* gene, next-generation sequencing

## Abstract

The main gene involved in gastric cancer (GC) predisposition is *CDH1*, the pathogenic variants of which are associated with diffuse-type gastric cancer (DGC) and lobular breast cancer (LBC). *CDH1* only explains a fraction (10–50%) of patients suspected of DGC/LBC genetic predisposition. To identify novel susceptibility genes, thus improving the management of families at risk, we performed a multigene panel testing on selected patients. We searched for germline pathogenic variants in 94 cancer-related genes in 96 GC or LBC Italian patients with early-onset and/or family history of GC. We found *CDH1* pathogenic variants in 10.4% of patients. In 11.5% of cases, we identified loss-of-function variants in *BRCA1*, *BRCA2*, *PALB2*, and *ATM* breast/ovarian cancer susceptibility genes, as well as in *MSH2*, *PMS2*, *BMPR1A*, *PRF1*, and *BLM* genes. In 78.1% of patients, we did not find any variants with clear-cut clinical significance; however, 37.3% of these cases harbored rare missense variants predicted to be damaging by bioinformatics tools. Multigene panel testing decreased the number of patients that would have otherwise remained genetically unexplained. Besides *CDH1*, our results demonstrated that GC pathogenic variants are distributed across a number of susceptibility genes and reinforced the emerging link between gastric and breast cancer predisposition.

## 1. Introduction

Gastric cancer (GC) is one of the most common cancers worldwide, ranking fifth for incidence and third for mortality [1]. Sporadic GCs are mainly of intestinal histotype (IGC) [2], they frequently occur at an old age (>65 years) and are associated with different environmental risk factors, including *Helicobacter pylori* infection, smoking, and a diet high in smoked and salted foods [3]. About 10% of GC patients cluster in families, likely due to low/medium-penetrance susceptibility variants or to shared environmental risk factors, or to a combination of both. About 1–3% of GCs can be considered hereditary, due to highly penetrant germline lesions in cancer-predisposition genes [4]. Hereditary GCs are mainly of diffuse histotype (DGC) [2], they are characterized by early-onset, and can be associated with different susceptibility genes [5].

The gene most frequently associated with hereditary cases is *CDH1* (OMIM *192090) [6], pathogenic variants of which are causative of the hereditary diffuse gastric cancer syndrome (HDGC, OMIM #137215), a dominant condition predisposing to both DGC and breast cancer of lobular histotype (LBC). *CDH1* encodes the transmembrane protein E-cadherin, which plays a key role in cell-cell adhesion and signal transduction, regulating cell differentiation and survival [6]. Since HDGC syndrome was first recognized in 1998 [7], about 150 *CDH1* variants have been reported, 80% of which are clearly pathogenic, while 20% remain of uncertain clinical significance (VUS: variants of uncertain significance) [5,8,9].

Selection criteria for genetic assessment of HDGC patients have been established and subsequently updated by the international gastric cancer linkage consortium (IGCLC) [10,11,12,13]. Selection is based on cancer histology (DGC and LBC), age of cancer onset, and number of affected subjects within pedigrees. By following guideline criteria for genetic testing, the detection rate of *CDH1* pathogenic variants is 30–50% in selected patients from populations with low GC incidence, while it drops down to 10–20% in countries (like Italy) with a medium-high incidence of GC [14,15]. The latter is likely due to inadequacy of criteria in discriminating inherited cases from familial clustering that frequently occurs in populations with high prevalence of GC risk factors. Although patient selection remains the relevant issue, a multimethod approach for *CDH1* testing is known to improve the variant detection rate, allowing identification of DNA sequence changes, large deletions, and gene expression defects [15].

Besides HDGC, a number of hereditary syndromes and corresponding genes are known to increase GC risk. These genes include *MLH1*, *MSH2*, *MSH6*, and *PMS2* (Lynch syndrome, LS), *TP53* (Li-Fraumeni syndrome, LFS), *APC* (Familial adenomatous polyposis, FAP, and gastric adenocarcinoma and proximal polyposis of the stomach, GAPPS), *MUTYH* (*MUTYH*-associated polyposis, MAP), *BMPR1A*, and *SMAD4* (Juvenile polyposis syndrome, JPS), *STK11* (Peutz-Jeghers syndrome, PJS), and *PTEN* (Cowden syndrome, CS) [13,16,17].

In the last few years, the availability of next-generation sequencing (NGS) has enabled screening for a number of genes on patients with suspected HDGC lacking *CDH1* pathogenic variants. By NGS-based approaches, GC risk-variants have emerged in *BRCA1*/*2*, *PALB2*, *ATM*, and *RAD51C* genes (typically associated with hereditary breast and ovarian cancer, HBOC), as well as in *CTNNA1, MAP3K6, MYD88*, and in other genes [9,18,19,20,21,22,23,24,25]. Although the exact contribution of some of these variants to GC development remains to be assessed by segregation analysis, by recurrence among HDGC families, and by consistent evidence from tumor samples, it is clear that NGS will progressively increase the number of genetic associations and expand our knowledge on GC missing heritability. Conversely, some recently highlighted variants raise the question of the clinical phenotype associated with each cancer susceptibility gene, creating uncertainty regarding appropriate surveillance and clinical management of variant carriers [26].

In this study, we aimed at identifying, by multigene panel (MGP) testing, genetic variants associated with GC and/or LBC in patients with suspected cancer predisposition. To our knowledge, this is the first NGS-based analysis on a large cohort of Italian patients.

## 2. Results

We analyzed a case series of 96 patients with a panel of 94 genes involved in cancer predisposition (Table 1).

All variants classified as pathogenic/likely-pathogenic based on the strict criteria we adopted (see variant classification in Materials and Methods) are listed in Table 2 and Table 3.

**Figure 1:**
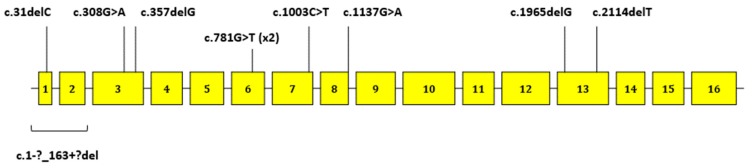
Schematic representation of the *CDH1* gene and localization of the nine pathogenic/likely-pathogenic variants identified in this work.

We identified nine *CDH1* pathogenic/likely-pathogenic variants in 10 out of 96 patients (10.4%). Four were frameshift deletions, three were nonsense variants (one found in to unrelated subjects), one was a synonymous variant affecting RNA splicing, and one was a gross deletion detected by Multiplex Ligation-dependent Probe Amplification (MLPA). Five out of nine pathogenic/likely-pathogenic variants had previously been reported [9,14,27,28,29,30,31,32,33,34,35,36,37], while four were novel. The nine variants were distributed along the entire gene (Figure 1). Among carriers, nine patients had DGC only (mean age: 39.9 years) and one had LBC only (52 years of age). *CDH1* molecular data, clinical features, and selection criteria of variant carriers are summarized in Table 2.

In 11 out of 96 patients (11.5%), we found loss-of-function variants in genes other than *CDH1*, including *ATM* (2 variants), *PALB2* (2 variants), *BRCA1*, *BRCA2*, *MSH2*, *PMS2*, *BMPR1A*, *PRF1*, and *BLM*. Four out of 11 variants were frameshift deletions (*ATM*, *BLM*, *PMS2*, *BRCA1*), one was a frameshift insertion (*ATM*), five were nonsense variants (*PRF1*, *PALB2*, *BRCA2*, *BMPR1A*), and one was a gross deletion (*MSH2*) detected by MLPA. Seven out of 11 variants had previously been reported [30,38,39,40,41,42,43,44], while four were novel. Five variant carriers had developed DGC (mean age: 54.2 years), one of whom after an LBC (50 years of age); three carriers had IGC (mean age: 57.0 years); one had bilateral LBCs (at 62 and 66 years of age), one had LBC at 61 years, and one had several GPs diagnosed at 52 years. Molecular data, clinical features, and selection criteria of variant carriers are summarized in Table 3.

The *BRCA1* variant was found in a subject with DGC and a family history of both GC and BC. The *BRCA2* pathogenic variant was found in two non-identical twins who both developed IGC at 60 years of age; their maternal cousin died of BC at less than 50 years of age. One *ATM* pathogenic variant was detected in an IGC patient with a strong family history of IGC and no BC cases in his family, while the second one was detected in an isolated patient with DGC at 32 years of age. One *PALB2* variant was carried by an LBC patient with GC and BC family history. The carrier of the other *PALB2* variant developed two LBCs, at 62 and 66 years of age; her sister and mother died of ductal BC at 55 years of age and of DGC at 52 years of age, respectively. On the whole, only *ATM* variant carriers (two out of six cases) had only GC and no family history of BC.

In our case series, the *MSH2* variant co-segregated with different cancers of the LS spectrum, being colorectal cancer predominant in the mutated family. On the contrary, the family of the *PMS2* variant carrier was characterized by GC development. The *BMPR1A* variant carrier (52 years old) had only developed gastric polyps; interestingly, this same phenotype was shared by his two sisters, in whom, however, we could not perform genetic testing.

The *BLM* variant carrier had developed both DGC and LBC, she was *BRCA1/2* negative, and showed a family history of GC and BC. Similarly, the *PRF1* variant carrier showed a family history of GC.

**Figure 2:**
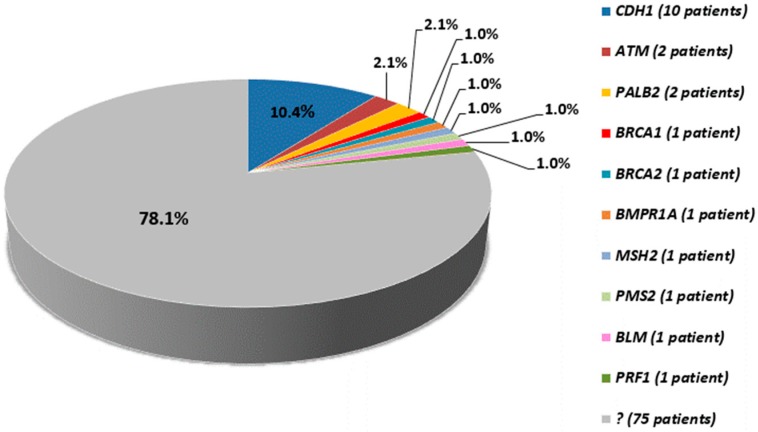
Pie chart showing the fraction of cases with/without pathogenic variants; the number of variant carriers is reported between brackets.

In the remainder of the tested cohort, i.e., 75 out of 96 patients (78.1%), we did not find any variants with clear-cut impact on gene function and clinical relevance (Figure 2).

To deeply investigate these last cases, we considered all variants identified by MGP testing. Globally, the 75 patients showed 7489 exonic variants. To exclude polymorphisms, we used allelic frequencies reported in 1000Genomes, Esp6500, and ExAC databases. The 271 variants with frequencies <1% or n/a, included: 93 (34.3%) synonymous base changes, 173 (63.8%) missense variants, three (1.1%) in-frame deletions, and two (0.7%) in-frame insertions.

We identified a total of 244 unique variants in 76 different genes. To assess their role in cancer development, we evaluated the 160 unique missense variants by using PolyPhen-2 HVAR and SIFT bioinformatics tools that predict functional impact and pathogenicity of human variants. Sixty-six out of 160 variants (41.3%) were classified as benign by both PolyPhen-2 HVAR and SIFT, 63 (39.4%) were discordantly classified, and 31 (19.4%) were classified as probably damaging by both bioinformatics tools (Table S1). Four out of the 31 variants classified as probably damaging (*MET*, *WRN*, *NBN,* and *TSC2* genes) were present in two patients (Table S1). Overall, these 31 variants were present in 28 patients, with 7/28 patients (BM58, BM61, BM67, BM75, BM93, BM118, BM122) carrying two different variants classified as probably damaging (Table S1). Within this group, 19/28 had DGC (mean age: 45.7 years), two of which had a previous or subsequent breast cancer; 2/28 had IGC (mean age: 61.5 years), one of which had a subsequent ovarian cancer and LBC; 4/28 had GC of unknown histotype (mean age: 55.3 years), one of which with bilateral LBCs; 3/28 had LBC (mean age: 50.0 years), one of which with a subsequent contralateral LBC.

## 3. Discussion

In the present work, we analyzed 96 Italian patients with suspected genetic predisposition to GC by sequencing 94 cancer-related genes.

*CDH1* was confirmed as the major GC predisposition gene, with a mutation frequency of 10.4%. All carriers of *CDH1* pathogenic/likely-pathogenic variants fell within the patient group selected according to criteria I-IV, i.e., criteria established by the IGCLC for HDGC [13] (see Materials and Methods). By only considering the 85 cases fulfilling criteria I-IV, the percentage of *CDH1* patients with pathogenic/likely-pathogenic variants was 11.8%.

For *CDH1* mutation carriers, the cumulative risk of developing GC by the age of 80 is 70% for men and 56% for women; women have an additional estimated risk of 42% of developing LBC by the age of 80 years [9]. Due to the high penetrance of *CDH1* pathogenic variants, the early age of onset, and the poor prognosis of DGC, prophylactic total gastrectomy is strongly recommended in mutation carriers. Indeed, the analysis of gastrectomy specimens performed over the years invariably revealed the presence of multiple foci of signet ring cell carcinoma (SRCC) [15,45,46]. Whenever prophylactic gastrectomy is not feasible, carriers should be offered appropriate endoscopic surveillance, as well as mammography surveillance of women. However, endoscopic surveillance for *CDH1* mutation carriers is largely ineffective, essentially due to the highly focal nature of HDGC, and only very experienced teams achieve a 50% rate of SRCC endoscopic detection [47]. In our survey, following the genetic test on consenting relatives of *CDH1*-mutation carriers, two subjects with a pathogenic variant decided to undergo prophylactic gastrectomy. In both cases, pathological analysis of gastric specimens detected GC microscopic foci, making the management of the disease easier and the outcome more favorable.

Regarding the 11 loss-of-function variants we found in genes other than *CDH1*, six were in genes associated with hereditary breast and ovarian cancer (HBOC), including *BRCA1*, *BRCA2*, *ATM,* and *PALB2*. *BRCA1* and *BRCA2* genes were found to be mutated in a single proband, while *ATM* and *PALB2* were both found to be mutated in two unrelated cases (6.3% of patients). 

Apart from the two non-identical twins with the *BRCA2* pathogenic variant, we could not perform co-segregation analysis for *BRCA1*, *ATM*, and *PALB2* genes due to compliance problems and difficulties in obtaining DNA samples. However, these genes have already been implicated in rare GC cases (of both intestinal and diffuse histotypes) by at least three independent studies aimed at identifying genetic predisposition to GC [9,21,24]. In addition, the *BRCA1* and *BRCA2* variants we identified have already proven to be disease-causative, at least in HBOC families [42,44].

Three out of 11 loss-of-function variants in genes other than *CDH1* were in *MSH2* and *PMS2* lynch syndrome (LS) genes and in *BMPR1A* juvenile polyposis syndrome (JPS) genes, accounting for 3.1% of patients in our case series. Although colorectal cancer (CRC) is predominant in LS and JPS, both syndromes have also been associated with an increased risk of GC [48,49]. In particular, LS mutation carriers have a 40–80% risk of CRC and a 11–19% lifetime risk of GC [50,51,52], while JPS mutation carriers have a 17–22% risk of CRC and a 10–21% lifetime risk of gastric and duodenal carcinoma [53,54,55]. 

We could not analyze gastric cancer tissue samples of *MSH2* and *PMS2* variant carriers for mismatch repair (MMR) deficiency. However, it is worth noticing that this somatic test could be useful to select GC patients with LS, similarly to what is performed on colorectal and endometrial cancers with the universal screening by MMR proteins immunohistochemical analysis [56]. On the other hand, by testing the *BMPR1A* variant carrier for germline mutations in promoter 1B of *APC* gene, we could exclude GAPPS [16,17], thus reinforcing the causal link between *BMPR1A* and gastric polyposis.

Finally, we identified two loss-of-function variants (2.1% of the patients) in *BLM* and *PRF1* genes that have been implicated in susceptibility to multiple cancers, mainly leukemias and lymphomas [57,58]. Both genes make biological sense for GC development: somatic mutations of *BLM* gene have already been identified in GC [59], and GC cases have been reported in families with *PRF1* germline mutations [60]. However, co-segregation data between cancers and germline variants are needed to definitively assess the role of *BLM* and *PRF1* genes in GC predisposition.

As far as the selection criteria is concerned, among the 85 patients fulfilling the IGCLC criteria for HDGC [13], 10 (12%) proved to carry *CDH1* pathogenic variants, while eight (9.4%) proved to carry pathogenic variants in other genes; of note, five of the latter patients showed pathogenic mutations in breast cancer genes (6%). Pathogenic variants in genes other than *CDH1* were also present in three out of 11 (27%) patients not fulfilling the IGCLC criteria. While on one hand, these findings indicate that IGCLC criteria are able to detect GC predisposition across a number of high penetrance genes, they also encourage the use of broader criteria for patients’ selection. In particular, our results suggest that families with a coexistence of GC (of any histotype) with breast or colon cancer should be tested with a panel including genes known to predispose to breast-ovarian and colorectal cancers, respectively.

In 75 patients (78.1%), we did not find any variants with clear clinical relevance. This fraction could be further reduced by analyzing a broader gene panel, including *CTNNA1* and other genes reported in rare cases of familial GC. At any rate, in 28/75 patients we identified rare missense variants (frequencies <1%) predicted to be damaging by two bioinformatics tools (Table S1): these patients showed clinical features similar to those of the other patients of the study cohort, in terms of cancer histotype and age of onset; accordingly, it is difficult to speculate if the identified variants are actually associated with an increased risk of GC. Besides refinements of criteria to improve patients’ selection, further studies should be performed to assess the functional impact of all these variants, including in vitro tests, tumor analysis, and segregation data.

## 4. Materials and Methods

### 4.1. Patients’ Selection

The patients (from different Italian regions) included in this study have been evaluated at the IRST genetic counselling service, upon request of a physician (general practitioner or medical specialist) and/or specific request of patients themselves. The medical geneticists of the service select patients eligible for genetic testing according to personal/family history and to established guidelines for cancer syndromes.

The patients of this study fulfilled the following criteria:

I: Two or more GC cases regardless of age, at least one confirmed DGC;
II: One case of DGC <40 years;
III: Personal or family history of DGC and LBC, one diagnosed <50 years;
IV: Bilateral LBC or family history of two or more cases of LBC <50 years;
V: GC ≤ 60 years with a family history of colorectal cancer;
VI: Two or more GC cases ≤ 60 years in first-degree relatives;
VII: Several gastric polyps ≤ 60 years with a family history of at least two GC cases.

Of note, Criteria I–IV are those of the HDGC updated guidelines established by the IGCLC [13]. Criterion V was adopted to select GC cases with suspected LS and criterion VI to investigate families showing an aggregation of GC of different types. Criterion VII was adopted to investigate patients with gastric polyps (GPs) and family history of GC, given that GPs and GCs can occur in polyposis syndromes (GAPPS, FAP, MAP, JPS, PJS, and CS).

Based on the above criteria, we selected 96 patients, including 79 with GC (57 with DGC, 14 with IGC, eight with GC of mixed or unknown histotype), 14 with LBC, and three with GPs.

### 4.2. Sample Collection and DNA Extraction

Peripheral blood was obtained from selected patients after informed consent and as approved by the institutional review board (CE IRST IRCCS-AVR, n.1952/2017). Blood was stored at −80 °C until genomic DNA was extracted. DNA was purified by QIAamp DNA Mini Kit (Qiagen, Hilden, Germany) and quantified using Qubit fluorometer (Thermo Fisher Scientific, Waltham, MA, USA) with Qubit dsDNA BR Assay Kit.

### 4.3. Multigene Panel (MGP) Testing

Sequencing libraries were created starting from 50 ng of genomic DNA, following the enrichment protocol TruSight Cancer (Illumina, San Diego, CA, USA) for simultaneous sequencing of 94 genes associated with a predisposition towards common and rare cancers (Table 1).

The MGP targets a total of 255 kb of the human genome, i.e., 1700 exons of the genes, as well as their flanking regions (on average 50 bp upstream and downstream each exon). 

Sequencing was performed by using the MiSeq platform (Illumina) with MiSeq Reagent Kit v2 configured 2 × 150 cycles, according to the manufacturer’s instructions.

### 4.4. Data Analysis and Variant Calling

The bioinformatics analysis of MGP results was performed with a customized pipeline [61].

Raw de-multiplexed reads from the MiSeq sequencer were aligned to the reference human genome (UCSC-Build37/hg19) using Burrows–Wheeler algorithm [62], running in paired-end mode. To ensure good call quality, to reduce the number of false positives and to identify variants, samples were analyzed with genome analysis toolkit GATK, version 3.2.2 [63]. Genomic and functional annotations of detected variants were made by Annovar [64]. Coverage statistics was performed by DepthOfCoverage utility of GATK. BASH and R custom scripts were used to obtain the list of low coverage (50×) regions per sample.

### 4.5. Additional Molecular Analyses

*CDH1* regions covered <50× were amplified by standard polymerase chain reaction (PCR) and PCR products were sequenced by using BigDye Terminator v.3.1 cycle sequencing kit (Thermo Fisher Scientific) on an ABI-3130 Genetic analyzer (Applied Biosystems, Foster City, CA, USA).

To identify the possible presence of *CDH1* extended deletions/duplications non-detectable by sequencing, samples were analyzed by MLPA method, by using P083-CDH1 kit (MRC Holland, Amsterdam, The Netherlands). Given the suspicion of LS, the two patients fulfilling criterion V were also tested with P003-MLH1/MSH2 and P072-MSH6 MLPA kits (MRC Holland), in order to identify possible deletions/duplications of *MLH1*, *MSH2*, and *MSH6* genes. All MLPA results were analyzed with Coffalyser software (MRC Holland).

### 4.6. Confirmation of Variants

All *CDH1* variants of classes 3–5 identified by MGP testing were confirmed by Sanger sequencing with the same protocol used for the uncovered regions.

*CDH1* and *MSH2* rearrangements identified by MLPA were confirmed through a second MLPA test.

All deleterious variants of classes 4–5 identified in genes other than *CDH1* were confirmed through a second NGS-based analysis.

### 4.7. Variant Classification

The identified genetic variants were divided into five classes according to the international agency for research on cancer (IARC) recommendations [65].

The classification of variants emerged from MGP testing was obtained by using the online databases LOVD CDH1 [66], dbSNP [67], and ClinVar [30].

Variants not included in any of these databases were classified on the basis of their characteristics: only variants producing premature stop codons and gross deletions were considered pathogenic (class 5) or likely-pathogenic (class 4) and classified in accordance with the guidelines of the American college of medical genetics (ACMG) [68] and the most recent guidelines on *CDH1* variant classification [26].

The potential impact of amino acid changes was assessed with PolyPhen-2 HVAR [69] and SIFT [70].

## 5. Conclusions

In conclusion, our results show that, in addition to *CDH1* genetic lesions, rare variants distributed across different genes can predispose to GC. Among these, there are also genes known to predispose to breast and ovarian cancer, thus reinforcing the emerging link between GC and BC predisposition. This last finding raises the question of clinical phenotypes associated with individual cancer susceptibility genes and adds a new challenge for management and appropriate surveillance in some families.

## Figures and Tables

**Table 1:** The 94 cancer predisposition genes included in the Trusight cancer panel.

*AIP*	*ALK*	*APC*	*ATM*	*BAP1*	*BLM*	*BMPR1A*	*BRCA1*	*BRCA2*	*BRIP1*
*BUB1B*	*CDC73*	*CDH1*	*CDK4*	*CDKN1C*	*CDKN2A*	*CEBPA*	*CEP57*	*CHEK2*	*CYLD*
*DDB2*	*DICER1*	*DIS3L2*	*EGFR*	*EPCAM*	*ERCC2*	*ERCC3*	*ERCC4*	*ERCC5*	*EXT1*
*EXT2*	*EZH2*	*FANCA*	*FANCB*	*FANCC*	*FANCD2*	*FANCE*	*FANCF*	*FANCG*	*FANCI*
*FANCL*	*FANCM*	*FH*	*FLCN*	*GATA2*	*GPC3*	*HNF1A*	*HRAS*	*KIT*	*MAX*
*MEN1*	*MET*	*MLH1*	*MSH2*	*MSH6*	*MUTYH*	*NBN*	*NF1*	*NF2*	*NSD1*
*PALB2*	*PHOX2B*	*PMS1*	*PMS2*	*PRF1*	*PRKAR1A*	*PTCH1*	*PTEN*	*RAD51C*	*RAD51D*
*RB1*	*RECQL4*	*RET*	*RHBDF2*	*RUNX1*	*SBDS*	*SDHAF2*	*SDHB*	*SDHC*	*SDHD*
*SLX4*	*SMAD4*	*SMARCB1*	*STK11*	*SUFU*	*TMEM127*	*TP53*	*TSC1*	*TSC2*	*VHL*
*WRN*	*WT1*	*XPA*	*XPC*						

**Table 2:** Carriers of *CDH1* deleterious variants.

Patient ID	Sex	Selection Criteria	Cancer	Age at Diagnosis	Gene	Exon	CDNA	Protein	IARC Class	DbSNP	ClinVar	Literature
BM112	F	II	DGC	37	*CDH1*	1–2	c.1-?_163+?del	p.?	5	−	−	Oliveira C. et al. 2009 [14]
BM73	F	III	LBC	52	*CDH1*	1	c.31delC	p.(Leu11Cysfs*45)	4	−	−	−
BM37	F	II	DGC	37	*CDH1*	3	c.308G>A	p.Trp103*	5	−	pathogenic	−
BM100	M	I	DGC	58	*CDH1*	3	c.360delG	p.(His121Thrfs*94)	4	−	−	−
BM81	F	II	DGC	18	*CDH1*	6	c.781G>T	p.Glu261*	5	rs121964873	pathogenic	Berx G. et al. 1995 [31]
BM115	F	II	DGC	31	*CDH1*	6	c.781G>T	p.Glu261*	5	rs121964873	pathogenic	Berx G. et al. 1995 [31]
BM60	M	II	DGC	39	*CDH1*	7	c.1003C>T	p.Arg335*	5	rs587780784	pathogenic	Jonsson B.A. et al. 2002 [32]
BM119	M	II	DGC	33	*CDH1*	8	c.1137G>A	p.Thr379=	4–5	rs587783050	pathogenic/likely pathogenic	Frebourg T. et al. 2006 [36]
BM74	M	I	DGC	59	*CDH1*	13	c.1965delG	p.(Met656Trpfs*3)	4	−	−	−
BM45	M	I	DGC	47	*CDH1*	13	c.2114delT	p.(Leu705Cysfs*17)	4	−	−	−

DGC: diffuse-type gastric cancer; LBC: lobular breast cancer. Selection criteria I–IV correspond to those established by the IGCLC for HDGC (see Materials and Methods).

**Table 3:** Carriers of deleterious variants in cancer-related genes.

Patient ID.	Sex	Selection Criteria	Cancer(s)	Age at Diagnosis	Gene	Exon	CDNA	Protein	IARC Class	DbSNP	ClinVar	Literature
BM10	M	V	IGC	57	*MSH2*	3	c.367-?_645+?del	p.?	5	−	pathogenic	Wijnen J. et al. 1998 [38]
BM90	M	I	DGC	73	*PMS2*	13	c.2182_2183delAC	p.(Thr728Serfs*7)	4	−	−	−
BM89	F	I	DGC	65	*PRF1*	3	c.1122G > A	p.Trp374*	5	rs104894176	pathogenic	Stepp S.E. et al. 1999 [39]
BM46	M	I + VI	IGC	54	*ATM*	10	c.1564_1565delGA	p.Glu522Ilefs*43	5	rs587779817	pathogenic	Lakin N.D. et al. 1996 [40]
BM76	F	II	DGC	32	*ATM*	14	c.2192dupA	p.(Tyr731*)	4	−	−	Saviozzi S. et al. 2003 [41]
BM38	M	VI	IGC	60	*BRCA2*	11	c.6037A > T	p.Lys2013*	5	rs80358840	pathogenic	Meindl A. et al. 2002 [42]
BM24	M	VII	GP	52	*BMPR1A*	3	c.34G > T	p.(Gly12*)	4	−	−	−
BM47	F	I	LBC, DGC	50, 54	*BLM*	11	c.2395delT	p.(Cys799Valfs*16)	4	−	−	−
A530	F	IV	LBC, LBC	62, 66	*PALB2*	4	c.535C > T	p.(Gln179*)	4	−	−	−
BM126	F	IV	LBC	62	*PALB2*	7	c.2718G > A	p.Trp906*	4–5	rs180177122	pathogenic/likely pathogenic	Casadei S. et al. 2011 [43]
BM110	F	I	DGC	47	*BRCA1*	7	c.406delA	p.Arg136Aspfs*27	5	rs886040196	pathogenic	Kluska A. et al. 2015 [44]

DGC: diffuse-type gastric cancer; IGC: intestinal-type gastric cancer; GP: gastric polyposis; LBC: lobular breast cancer. Selection criteria I–IV correspond to those established by the IGCLC for HDGC (see Patients’s Selection in Materials and Methods).

## Supplementary Materials

The following are available online at https://www.mdpi.com/2072-6694/11/9/1340/s1, Table S1: List of the 31 missense variants classified as probably damaging by both SIFT and PolyPhen2 identified in our case series and patients’ characteristics.
