# Supplementary material for: Multigene Panel Testing Increases the Number of Loci Associated with Gastric Cancer Predisposition
Source: Cancers (Basel). 2019 Sep 11;11(9):1340. doi: 10.3390/cancers11091340 (PMC6769562; doi:10.3390/cancers11091340)
Supplement: Supplementary file 1 [file cancers-11-01340-s001.pdf]

Supplementary Materials: Multigene Panel Testing Increases the Number of Loci Associated with Gastric Cancer Predisposition

Gianluca Tedaldi, Francesca Pirini, Michela Tebaldi, Valentina Zampiga, Ilaria Cangini, Rita Danesi, Valentina Arcangeli, Mila Ravegnani, Raefa Abou Khouzam, Chiara Molinari, Carla Oliveira, Paolo Morgagni, Luca Saragoni, Maria Bencivenga, Paola Ulivi, Dino Amadori, Giovanni Martinelli, Fabio Falcini, Guglielmina Nadia Ranzani and Daniele Calistri

Table S1. List of the 31 missense variants classified as probably damaging by both SIFT and PolyPhen2 identified in our case series and patients' characteristics.

Gene	Exon	cDNA	Protein	dbSNP	ClinVar	SIFT	Polyphen2 HVAR	Patient ID	Sex	Selection criteria	Cancer(s)	Age at Diagnosis
FH	2	c.234C>A	p.Asn78Lys	-	-	D	D	BM32	F	I	DGC	49
EPCAM	6	c.605A>C	p.Lys202Thr	-	-	D	D	BM67	F	I	DGC	41
PMS1	3	c.224C>T	p.Thr75Ile	rs61756360	-	D	D	BM31	F	I	DGC	47
FANCD2	3	c.195G>C	p.Gln65His	rs36084488	VUS/Benign	D	D	BM71	M	II	DGC	35
GATA2	5	c.1010G>A	p.Arg337Gln	-	-	D	D	BM67	F	I	DGC	41
KIT	21	c.2867G>A	p.Arg956Gln	rs139694927	VUS	D	D	BM85	M	I	IGC	66
PMS2	2	c.52A>G	p.Ile18Val	rs63750123	VUS/Likely benign/Benign	D	D	BM56	F	I	DGC	55
MET	14	c.2975C>T	p.Thr992Ile	rs56391007	VUS/Likely benign/Benign	D	D	BM93	M	I	DGC	65
MET	14	c.2975C>T	p.Thr992Ile	rs56391007	VUS/Likely benign/Benign	D	D	BM50	F	IV	LBC, LBC	53, 58
EZH2	19	c.2151G>C	p.Gln717His	-	-	D	D	BM62	M	I	DGC	45
WRN	14	c.1717A>G	p.Thr573Ala	rs150148567	VUS/Likely benign	D	D	BM58	M	I	DGC	54
WRN	14	c.1717A>G	p.Thr573Ala	rs150148567	VUS/Likely benign	D	D	BM75	F	I	IGC, OC, LBC	57, 60, 72
WRN	17	c.1909C>T	p.Arg637Trp	rs148286708	VUS	D	D	BM58	M	I	DGC	54
WRN	30	c.3523C>A	p.Pro1175Thr	-	-	D	D	BM84	F	I	LBC, DGC	61, 66
NBN	3	c.283G>A	p.Asp95Asn	rs61753720	VUS/Likely benign/Benign	D	D	BM41	F	III	LBC	45
NBN	3	c.283G>A	p.Asp95Asn	rs61753720	VUS/Likely benign/Benign	D	D	BM96	F	II	DGC	33
RET	11	c.1946C>T	p.Ser649Leu	rs148935214	VUS/Likely benign/Benign	D	D	BM69	F	I	GC	77

RET	11	c.1997A>T	p.Lys666Met	rs377767439	VUS	D	D	BM48	F	I	GC	47
BMPR1A	11	c.1243G>A	p.Glu415Lys	rs140592056	VUS/Likely benign/Benign	D	D	BM79	F	III	LBC	52
EXT2	5	c.889C>T	p.Arg297Cys	rs146098187	-	D	D	BM93	M	I	DGC	65
CEP57	3	c.333G>C	p.Gln111His	rs117321017	Benign	D	D	BM75	F	I	IGC, OC, LBC	57, 60, 72
BRCA2	14	c.7225C>T	p.Pro2409Ser	-	-	D	D	BM72	M	II	DGC	25
ERCC5	14	c.2890C>T	p.Arg964Trp	rs574826021	VUS	D	D	BM61	F	I	DGC	49
FANCM	20	c.4931G>A	p.Arg1644Gln	rs138151018	Likely benign/Benign	D	D	BM61	F	I	DGC	49
FANCI	18	c.1813C>T	p.Leu605Phe	rs117125761	Likely benign/Benign	D	D	BM122	M	I	GC	59
BLM	12	c.2474C>T	p.Pro825Leu	rs749632465	VUS	D	D	BM118	F	I	DGC	54
TSC2	17	c.1747G>A	p.Ala583Thr	rs1800729	Benign/Likely benign	D	D	BM21	F	I	DGC	47
TSC2	17	c.1747G>A	p.Ala583Thr	rs1800729	Benign/Likely benign	D	D	BM63	F	I	DGC, DBC	41, 50
SLX4	6	c.1192C>T	p.Arg398Trp	rs138799572	VUS	D	D	BM114	F	IV	LBC, GC, LBC	36, 38, 52
ERCC4	7	c.1135C>T	p.Pro379Ser	rs1799802	VUS/Likely benign	D	D	BM121	F	I	DGC	40
ERCC4	11	c.2117T>C	p.Ile706Thr	rs1800069	VUS	D	D	BM117	F	II	DGC	36
FLCN	6	c.503G>A	p.Arg168His	-	-	D	D	BM118	F	I	DGC	54
NF1	34	c.4526G>A	p.Arg1509His	rs546073780	VUS	D	D	BM124	M	I	DGC	51
RAD51D	10	c.932T>A	p.Ile311Asn	rs145309168	VUS/Likely benign	D	D	BM122	M	I	GC	59
RHBDF2	5	c.478C>T	p.Arg160Cys	rs751482282	VUS	D	D	BM120	F	II	DGC	36

VUS: variant of uncertain significance; D: damaging; GC: gastric cancer; DGC: diffuse-type gastric cancer; IGC: intestinal-type gastric cancer; LBC: lobular breast cancer; DBC: ductal breast cancer; OC: ovarian cancer. Selection criteria I–IV correspond to those established by the IGCLC for HDGC (see Materials and Methods).

© 2019 by the authors. Licensee MDPI, Basel, Switzerland. This article is an open access article distributed under the terms and conditions of the Creative Commons Attribution (CC BY) license (http://creativecommons.org/licenses/by/4.0/).
